# Characterization of Artificial Stone Produced with Blast Furnace Dust Waste Incorporated into a Mixture of Epoxy Resin and Cashew Nut Shell Oil

**DOI:** 10.3390/polym15204181

**Published:** 2023-10-21

**Authors:** Tatiane Brito Perim, Elaine Carvalho, Gabriela Barreto, Thaís Leal da Cruz Silva, Sérgio Neves Monteiro, Afonso Rangel Garcez de Azevedo, Carlos Maurício Fontes Vieira

**Affiliations:** 1 Advanced Materials Laboratory (LAMAV), State University of Northern Fluminense—UENF, Av. Alberto Lamego, 2000, Campos dos Goytacazes 28013-602, RJ, Brazil; tatianeperim@hotmail.com (T.B.P.); elainesanttos@yahoo.com.br (E.C.); gabibarreto93@gmail.com (G.B.); 202111220042@pq.uenf.br (T.L.d.C.S.); vieira@uenf.br (C.M.F.V.); 2 Department of Materials Science, Instituto Militar de Engenharia–IME, Praça General Tibúrcio, 80, Praia Vermelha, Urca, Rio de Janeiro 22290-270, RJ, Brazil; snevesmonteiro@gmail.com; 3 Civil Engineering Laboratory (LECIV), State University of the Northern Rio de Janeiro—UENF, Av. Alberto Lamego, 2000, Campos dos Goytacazes 28013-602, RJ, Brazil

**Keywords:** artificial stone, blast furnace dust waste, epoxy resin

## Abstract

The demand for materials with improved properties and less negative impact on the environment is growing. Artificial stones are examples of these materials produced with up to 90% of particulate material joined by a binder. This article evaluates the physical and mechanical properties of two artificial stones produced with processing steel residue (blast furnace dust waste) and quartz powder. Two binders were used: pure epoxy resin, denoted as ASPB100, or a mixture of 70 wt% epoxy resin with 30 wt% cashew nut shell oil, denoted as ASPB7030. The process took place under vibration, compression (3 MPa/20 min and 90 °C) and vacuum (80 Pa). ASPB100 showed water absorption of 0.07%, while for ASPB7030, it was 0.54%. They were classified as having high mechanical strength associated with bending stress values equal to 32 and 25 MPa, respectively. Stain resistance indicated that both artificial stones had their stains removed with the tested cleaning agents. In this way, the novel artificial stones produced are sustainable alternatives for the application of blast furnace waste and cashew nut shell oil, reducing their negative impacts on the environment.

## 1. Introduction

Artificial stone is defined as a composite material made from a mixture of stone aggregates and other minerals bonded together with a polymeric resin, which allows a material with mechanical properties close to or superior to natural stones. Aggregates normally represent between 90 and 94 wt% of artificial stones [1].

Faced with the growing demand for diversified materials with improved physical and mechanical properties, artificial stones have caught the interest of consumers due to the advantages they have over natural stones, such as the minimum amount of pores, which contributes to a more compact and durable material. Artificial stones might have better mechanical resistance, lower density (due to the polymeric matrix) and the possibility of being manufactured from industrial waste, such as those from the ornamental stone industry as well as glass and steel industries, among others, with different textures, colors and shapes [2,3,4].

Due to the advantages presented by artificial stones, they have been widely used in decoration and construction. Although they originated in the 1960s in Italy, artificial stones became popular in the rest of Europe and North America only in the 1990s [4].

Owing to the control of the process and the inputs used in production, this material has a slightly higher market price than natural stones. The average price of artificial stones is currently USD 540/t, while for natural stones, this is USD 527/t [5].

Today, the artificial stone industry’s challenge is to make this material increasingly sustainable, economically viable and with improved mechanical properties, thus leading producers to seek alternative fillers to replace conventional aggregates. There are studies reporting the use of different solid wastes as mineral fillers in the production of artificial stones. Lee et al. [6] and Peixoto et al. [7] used waste glass. Sarami and Madavian [8] used travertine mud, Agrizzi et al. [9] added quartzite residue in a polyurethane matrix, Silva et al. [10] used marble residue in an epoxy matrix and Carvalho et al. [11] used as their residue an electrostatically precipitated powder obtained from the initial sintering stage of an integrated steelmaking plant.

The addition of mineral filler in the production of polymeric composites contributes to an improvement in mechanical resistance. Anastasios et al. [12] added fly ash powder in various contents and found an increase in mechanical resistance as the ash content increased. In this way, the reuse of industrial waste for the development of artificial stones can be a solution to reduce the cost of this material, in addition to avoiding illegal disposal of solid waste, generating jobs and boosting the economy [13,14,15].

Other additives such as natural fibers also bring improvements in mechanical properties. Rajawat et al. [16] produced composites by adding basalt fibers to marble waste and obtained an improvement in mechanical properties when compared to composites without adding the fiber. In addition to mechanical properties, other factors can also be improved through additives, such as combustion inhibitors, which help to reduce the combustibility of the polymer composite [17].

Polymer resin has the advantage of being lighter, thus reducing transportation costs; however, degradation in external environments is a disadvantage. This disadvantage can be improved through additives that act as inhibitors of its accelerated degradation, but more studies in this area are required [18].

As industrialization increases worldwide, wastes are becoming a modern environmental problem. Poor handling intensifies their improper disposal, thus jeopardizing landscapes, fauna, flora and populational health and leading to air and soil contamination. Hence, there is an urgent need to propose new sustainable strategies aiming to lessen these impacts through more effective waste management [19].

An alternative path to reduce the amount of improperly discarded waste while enhancing the mechanical properties of a commercial material is to produce artificial stones based on waste materials, which can be achieved by replacing part of the raw material aggregates (such as quartz) with these wastes. In this scenario, siderurgic waste has potential to be employed in artificial stone’s development since it has a chemical composition close to that of the conventional aggregates. In particular, the presence of metallic oxides, such as iron, contributes to increasing the mechanical strength of the final product [20].

Despite its economic importance, the steelmaking industry is a major waste generator by virtue of each stage of the siderurgic production process. According to the Steel Institute, in 2018, for each 1 ton of crude steel produced, 628.5 kg of waste and co-products was generated. Notwithstanding its 85% reuse potential, the remaining 15% still does not have an adequate destination, thus lingering as stock or discarded in industrial landfills [21].

Another of the artificial stone’s development challenges is the polymeric resin. Currently, the industry uses mostly epoxy or polyester, both derived from petroleum. Seeking bio-based resins as a substitute to make the material more sustainable, this study used a mixture of 70 wt% epoxy resin with 30 wt% cashew nut shell oil (CNSL). CNSL is an inedible agricultural waste widely available, with a production in the order of 4,450,000 tons per year. Cardanol is a CNSL major constituent and is characterized by a phenol ring connected to C15 alkyd chains at the meta position, which have different degrees of unsaturation. The unique structure of cardanol makes this material a promising candidate for a variety of applications and for many routes of chemical modifications, exhibiting excellent thermal and mechanical properties owing to its unique structural features [22,23].

Unlike previously published studies, this work produced an environmentally friendly artificial rock by modifying the binder through the addition of CNSL to the epoxy resin. This work’s objective was to produce and characterize a novel artificial stone based on blast furnace dust waste and conventional aggregate as fillers agglomerated by a mixture of epoxy resin with CNSL. Characterization was performed by evaluating the physical, chemical and mechanical properties of the artificial stones produced.

## 2. Materials and Methods

The blast furnace dust was supplied by ArcelorMittal, located in Serra, Espírito Santo, Brazil. The other congregate mineral material, quartz sand, was supplied by EcologicStone, located in Cachoeiro de Itapemirim, Espírito Santo, Brazil. Both wastes were applied to manufacture the artificial stones proposed. The quartz sand was supplied as particles of both medium and coarse granulometry, ranging from 2 to 0.42 mm and 0.42 to 0.075 mm. The blast furnace waste was supplied as fine granulometric particles of less than 0.075 mm.

As the binder, we used the epoxy resin type diglycidyl ether of bisphenol A (DGEBA) mixed with the triethylenetetramine (TETA) hardener, both supplied by Epoxyfiber, Brazil. The supplier indicated the density of the epoxy was 1.15 g/cm^3^.

The CNLS was supplied by Resibras, Fortaleza, Ceará, Brazil.

### 2.1. Materials’ Characterization

The blast furnace dust waste was subjected to an X-ray fluorescence (XRF) analysis for chemical characterization using a wavelength distribution XRF Spectrometer (model: S8 Tiger, brand: Bruker, anode composition: Rh (rhodium), max. 1 kW, voltage 20–50 kV, current: 5–50 mA). The mineralogical composition was studied in a SHIMADZU diffractometer operating with copper radiation (Cu-Kα) and a 2θ scanning ranging from 5° to 90°.

Three compositions were prepared for the epoxy resin: CNLS: EPOJU8020 (80–20 wt%), EPOJU7030 (70–30 wt%) and EPOJU6040 (60–40 wt%). In order to evaluate their mechanical properties, 3-point bend tests were performed as per the UNEEN ISO 178 standard (for plastics) [24], using the operation mode of an Instronmachine, model 5582, operating at a 5 mm/min^−1^ rate. The composition with the highest amount of CNLS presented the best bend strength and was thus chosen for the manufacturing of the artificial stones.

### 2.2. Determination of the Highest Packaging Granulometric Composition

**Figure 1:**
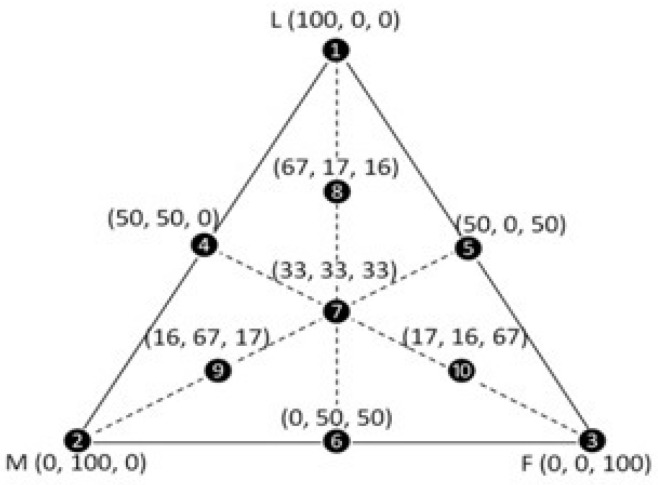
Ternary diagram with the 10 mixtures based on the SLD complete cubic model, showing the amounts (wt%) of large (L), medium (M) and fine (F) particles [27].

Ten different particle size compositions were proposed using three particle sizes: large (L), medium (M) and fine (F). Figure 1 displays a complete ternary diagram developed in the experimental numeric modeling grid Simplex Lattice Design (SLD) [25] to obtain the highest packaging granulometric composition. Each vertex of the triangle corresponds to 100% of the large (L), medium (M) and fine (F) particles. The distinct fractions of the mixtures are represented by the other points of the triangle.

The mixture with the highest packing was determined as per the ABNT/MB-3388—“Determination of the minimum index void ratio of non-cohesive” [26]. Each composition was tested three times for statistical validation. All the mixtures, one at a time, were placed in a steel vessel and left vibrating for 10 min under a load of 10 kg. The mixtures were then weighed and their vibrated densities determined.

The data obtained above the underwent analysis of variance (ANOVA) of the completely randomized design (CRD) (*p* ≤ 0.05) to corroborate the statistical significance. Then, the Tukey test (*p* ≤ 0.05) was performed to compare the averages and verify the one with the best results. All calculations were performed using Microsoft Excel from Office, 2010.

With the highest package composition vibrated density, it is possible to calculate the void volume (*VV*) of the mixture, using [28]:

(1) $$VV\%=\left(1-\frac{\rho_{PA}}{\rho_{Q}}\right)\times 100$$

where:

*VV*% = Void volume present in the mixture of particles;

$\rho_{PA}$ = Apparent density of particles, calculated using the packaging method;

*ρ_Q_* = Mineral charge density, calculated via pycnometry;

After that, to determine the minimum content of resin to fill the void volume and, therefore, produce the artificial stones, Equation (2) was used:

(2) $$MAR\%=\frac{VV\%\times \rho_{resin}}{VV\%\times \rho_{resin}+\left(100-VV\%\right)\times \rho_{Q}}$$

where:

*MAR*% = Minimum amount of resin to fill the void volume;

VV% = Void volume present in the mixture of particles;

$\rho_{resin}$ = Epoxy resin density;

*ρ_Q_* = Mineral charge density, calculated using pycnometry.

In this work, an 18% *MAR* of epoxy resin was calculated to fill the void volume between the waste mixture particles.

### 2.3. Production of Artificial Stones

Artificial stones were produced with epoxy resin and CNSL, in the composition determined via ANOVA and the resin content determined via MAR in Equation (2). Stones sized 100 × 100 × 15 mm were processed using vacuum, vibration and compression methods, with pure epoxy resin (ASPB100) and with 70 wt% epoxy resin and 30 wt% CNLS (ASPB7030). The stones were developed based on blast furnace dust waste and quartz sand.

Initially, the waste was dried in an oven at 100 °C to remove moisture, and then it was weighed and placed inside the mixer. The resin and the CNLS were added to the mixer and, after homogenizing, the resulting mass was deposited in the mold on a vibrating table. The vibration and vacuum were important to ensure that all mass was well spread in the mold cavity and avoid leaving air bubbles trapped in the mass. The mold was then placed in a hydraulic press (brand Marcone model MA 098-A) for 20 min while subjected to a compression under a pressure of 3 MPa, and heated at 90 °C.

The mold was disconnected from the vacuum system and cooled to room temperature (RT). The stones were removed and put in an oven at 90 °C for post-curing. At the end, they were sanded and cut according to the sizes recommended for each test.

### 2.4. Characterization of Artificial Stone Plates

The values of density, water absorption and apparent porosity were obtained from the standard tests according to the ABNT/NBR standard [29,30]. For each condition, 10 specimens were made with dimensions of 50 × 50 × 10 mm.

Ten prismatic specimens, cut from the ASPB100 and ASPB7030 stones, sized with dimensions of 10 × 100 × 25 mm, were three-point bend tested in a model 5582 Instron machine under a 0.25 mm/min loading rate, 100 KN load cell and 80 mm two-point distance, following the NBR 15845-6 standard, to evaluate the maximum bending stress [29,31].

Wear resistance was measured in terms of thickness loss with the Amsler wear test (in Amsler equipment branded MAQTEST). Two samples measuring 70 × 70 × 40 mm were subjected to wear on a 500 and 1000 m circular running track, respectively, according to the ABNT/NBR standard [32].

The microstructure of the fracture region after bending tests was evaluated by using scanning electron microscopy (SEM) for the analysis of the adhesion of particles to the DEGBA-TETA system. The equipment used was the SSX-550 SHIMADZU model operated by using secondary electrons.

Stain resistance was tested according to an adaptation of the NBR 10545-14 standard [33]. This test verifies the action of penetrating agents (green Cr_2_O_3_ and red Fe_2_O_3_), an oxidizing agent (iodine) and a film-forming agent (olive oil). Domestic products such as wine, ketchup, mustard, lemon and coffee were also used. For each coloring agent, a specimen was tested and the staining agent remained on its surface for 24 h. The material was then classified according to the ease of stain removal.

To remove stains, the specimens were subjected to the following cleaning procedures: (i) washing with hot water; (ii) manual cleaning process with lighter cleaning products; (iii) cleaning the surface with a strong cleaner; and (iv) immersion in acid solution. This test aims to identify the applicability of the artificial stone in places such as a kitchen, bathroom or office tops [7].

## 3. Results

### 3.1. Materials’ Characterization

According to Table 1, the blast furnace dust waste had a chemical composition with more than 70% iron oxide, followed by quartz and calcium. This result was expected since this waste was obtained from cleaning the gas generated in a steel industry blast furnace during the reduction of ore to pure iron.

**Figure 2:**
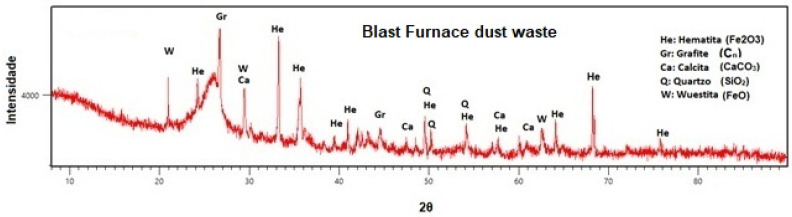
XRD analysis of blast furnace dust waste.

The results of the mineralogical analysis in Figure 2 confirm the chemical analysis, as many minerals formed by iron oxide are present, such as hematite (Fe_2_O_3_) and wustite (FeO). Furthermore, iron-based minerals like quartz (SiO_2_), calcite (CaCO_3_) and graphite (C_n_) are also present.

**Figure 3:**
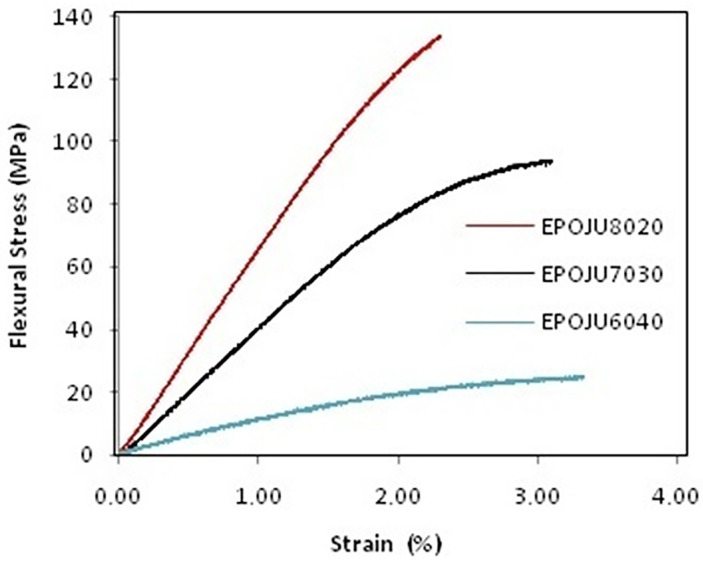
Flexural rupture strain for EPOJU8020, EPOJU7030 and EPOJU6040.

Figure 3 shows the bending stress vs. strain curves of the resin compositions tested with epoxy and CNSL. The composition that revealed the highest mechanical resistance was EPOJU8020, with 138 ± 2 MPa, followed by EPOJU7030 with 98 ± 3 MPa and EPOJU6040 with 20 ± 2 MPa. The results showed that the higher the epoxy resin content, the greater the mechanical resistance. Agrizzi et al. [9] evaluated the mechanical strength of pure epoxy resin and found it to be 93.6 ± 4.7 MPa. As goal in the present study was to insert the maximum CNSL possible, and the sample with the highest CNSL content displayed the highest mechanical resistance, the composition of EPOJU7030 with 70% epoxy and 30% CNSL was adopted to produce the artificial stone.

### 3.2. Determination of the Highest Packing Density

Table 2 presents the values obtained through the SLD method for testing the average vibrated density of mixtures of blast furnace dust waste and quartz sand waste, according to the compositions displayed in Figure 1.

As an important parameter, the vibrated density average data were treated with ANOVA considering a CRD performed with 95% confidence level (*p* ≤ 0.05), with subsequent Tukey test average results. Analyzing the results obtained in Table 3 and Table 4, it is possible to verify that the treatments studied presented statistical differences, meaning that among the 10 mixtures, at least two were differentiated.

The Tukey test was performed in order to differentiate corresponding results and we found that mixtures 4 and 8 possessed higher densities and differed statistically from each other. Since mixtures involving non-spherical particles have an enormous amount of possible particle shapes, leading to an infinity of particle combinations, it is hard to develop a model able to predict their behavior. The only accurate prediction is that the more non-spherical particles become, the lesser the packing density and other related properties.

In this study, it was decided to use composition 8 due to the presence of blast furnace dust waste since mixture 4 was composed only of quartz sand. Table 5 displays the particulate composition and the minimum amount of resin (calculated using Equation (2)) adopted for the production of ASPB100 and ASPB7030.

### 3.3. Physical Properties

Table 6 shows the ASPB100 and ASPB7030 physical properties.

The densities of ASPB100 and ASPB7030 were 2.29 and 2.21 g/cm^3^, respectively. As can be seen in Table 6, the artificial stone with CNLS in its composition had a lower density. This can be attributed to the density of CNSL, which is 0.97 g/cm^3^ [34], contrasting with the epoxy resin’s density of 1.15 g/cm^3^, making the stone lighter. It is important to evaluate the density since the higher its value, the better the particle/matrix adhesion, diminishing the occurrence of voids. Polymer-based matrices have the advantage of being lighter, thus reducing logistical costs [35].

Carvalho et al. [36] produced an artificial stone based on steelmaking waste using vacuum, vibration and compaction parameters. The material’s densities were found to vary from 2.68 to 2.73 g/cm^3^.

Sarami and Mahdavian [8] developed artificial stones with 85–90 wt% travertine marble waste and polyester resin, obtaining densities between 1.6 and 3.1 g/cm^3^ for their materials. They concluded that the addition of resin favors the density, up to a limit, since the artificial stone developed with 85 wt% had a density around 3.1 g/cm^3^ and the one with 90 wt% had 2.6 g/cm^3^.

As for the water absorption, ASPB7030 had the value of 0.54%, greater than that of 0.07% for ASPB100. Water absorption is related to the impermeability of the material’s surface, a highly relevant factor due to its applicability in humid environments.

Carvalho et al. [11] produced an artificial stone with steelmaking waste and epoxy resin with 0.24% the water absorption. It is worth noting that the addition of CNSL increased the water absorption and porosity of the produced stone. A higher resin content favors better physical properties, as the resin is responsible for providing efficient adhesion between the particles and also can penetrate between the interstices, eliminating the porosity of artificial stones [10,11,12,13,14,15,16,17,18,19,20,21,22,23,24,25,26,27,28,29,30,31,32,33,34,35].

Chiodi and Rodriguez [37], in their “Guide to the Application of Coating Stones”, classified quartzites with water absorption under 0.1% as materials with very low absorption, which reinforces the good performance of both developed stones, with emphasis on ASPB100.

### 3.4. Mechanical Properties

**Figure 4:**
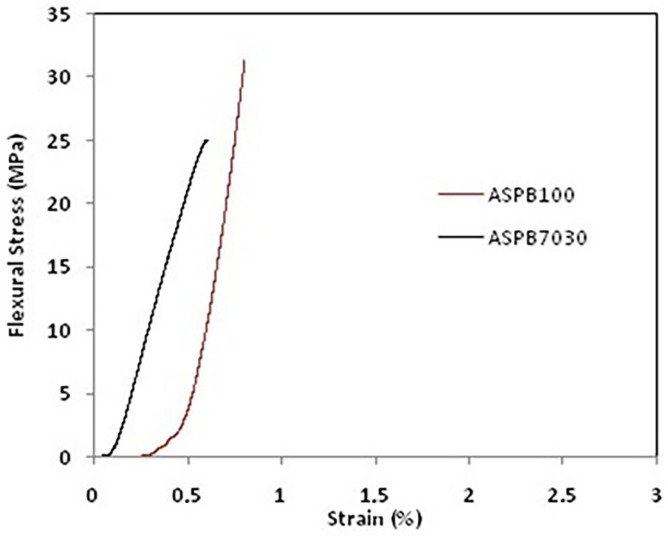
Three-point flexural strength of ASPB100 and ASPB7030.

Figure 4 shows the three-point bend stress vs. strain curves for ASPB100 and ASPB7030.

Figure 4 shows that ASPB100 and ASPB7030 had 32 and 25 MPa mechanical strength, respectively. These results were expected since it is known that ASPB7030 has higher water absorption and porosity, properties that influence the mechanical properties.

Following Chiodi and Rodriguez’s [37] guidelines, ASPB100 and ASPB7030 can be applied as coatings and classified as materials of very high strength for that application since both meet the parameter they established of a bending strength above 20 MPa.

Agrizzi et al. [7] produced an artificial stone based on 85 wt% quartzite waste and a polyurethane resin from a natural source that displayed 10.77 ± 0.64 MPa of flexural strength. Likewise, Gomes et al. [37] also produced artificial stone with a polyurethane resin and 85 wt% granite waste, and their material had 18.10 ± 1.62 MPa mechanical strength.

When compared to these previous studies, in which the authors also developed artificial stones with resins from natural sources, one can observe that both artificial stones in this study had satisfactory mechanical properties. This can be attributed to the molecular interconnections between the matrix and the agglomerates, since low dispersion indicates a considerable mechanical stability of the artificial material produced [31].

The addition of a natural oil to the polymeric matrix did not significantly affect the mechanical resistance; there was a decrease of approximately 20%, which is an advantage as, in addition to adding value to a residue, it reduces the amount of pure epoxy resin added, which can contribute to reducing the final price of the stone.

**Figure 5:**
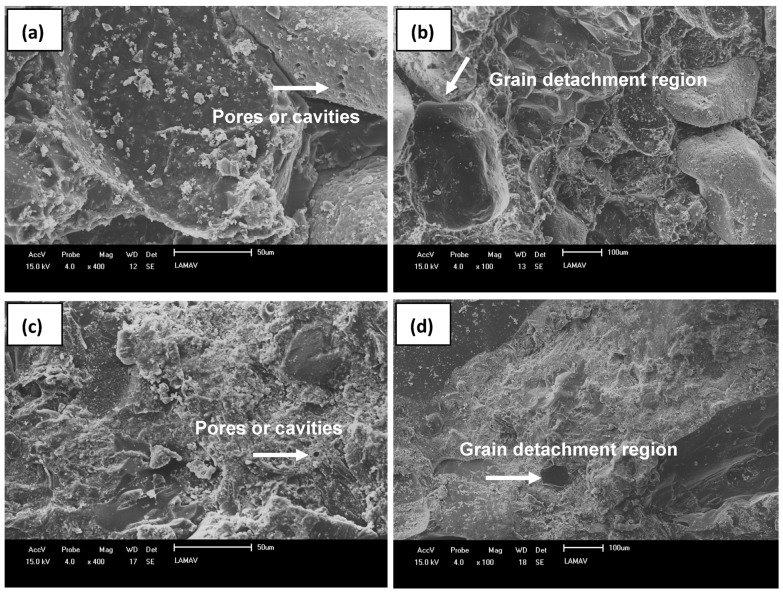
SEM of fractured region after 3-point bend strength tests: ASPB7030, (**a**) 400× and (**b**) 100×; ASPB100, (**c**) 400× and (**d**) 100×.

SEM micrographs of the fractured region after the three-point flexural strength test are displayed in Figure 5.

Through Figure 5, it is possible to confirm the good interaction between the agglomerate particles and the resin in both artificial stones, indicating that the mixture was well homogenized. In addition, the low amount of pores can be noted, which appeared in isolation in Figure 5a,c. The use of vacuum and vibration during the manufacturing process is likely to have contributed to the removal of air and consequently to a decrease in the amount of empty space between particles [31].

In Figure 5b, it is possible to identify regions of grain detachment. However, these cannot be seen for ASPB100. The detachment of grains during fracture in ASPB7030 can be associated with the CNSL content used in this material, which contributed to a poorer adhesion of the aggregates at the interface. In fact, the function of the polymeric resin is to join the particles, filling the empty spaces for them, but not to become a stress concentrator and form preferential paths to failure.

As ASPB7030 has a lower epoxy resin content, the particles were not as interconnected, contributing to the detachment of the grains when flexural stress was applied; however, the mechanical properties were still satisfactory.

Table 7 displays the results obtained in the abrasive wear test for both artificial stones produced. As per Chiodi Filho and Rodrigues’ [37] classification, artificial stones meant to be applied as pavement are classified in terms of thickness loss after the Amsler wear test as follows: high traffic floor (<1.5 mm), medium traffic floor (<3 mm) and low traffic floor (<6 mm).

Based on the technological parameters outlined by Chiodi Filho and Rodrigues [37], one can observe that ASPB100 meets the requirements to be applied as coating on floors subjected to high traffic due to the efficient rearrangement in the structure and texture of the minerals during the agglutination of the particles in the polymer matrix [7,37]. On the other hand, ASPB7030 had a 2.09 mm thickness loss on the same running track and, therefore, is ideal to be applied in floor covering subjected to medium traffic.

The loss of thickness of ASPB7030 was revealed by previous analyses of physical properties, such as water absorption and porosity, and micrographs, which included regions of grain detachment in the region under tension, demonstrating a low efficiency of particle adhesion in the polymer matrix due to the presence of natural oil. However, the rock is still within the standard established by Chiodi Filho and Rodrigues [37].

### 3.5. Chemical Properties

**Figure 6:**
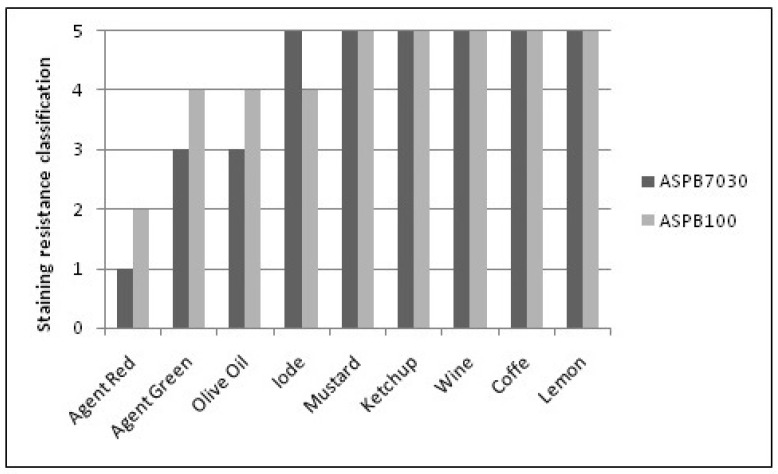
Classification regarding stain resistance of ASPB7030 and ASPB100.

Figure 6 displays a graph containing the stain resistance test results. The resistance to staining is evaluated by the degree of difficulty in removing the staining agent after the cleaning procedures contained in the NBR 10545-14 standard [33]. The number 5 represents the easiest stain removal, while the number 1 represents the hardest one; that is, stones classified as number 5, for some staining agents, did not have their stain removed, even after going through all the cleaning procedures indicated by the standard.

In Figure 6, one can observe that the majority of the staining agents were easily removed (number 5) for both artificial stones developed. Still, the red agent could not be removed for ASPB7030 after all the cleaning steps. This may be associated with the greater number of pores in this material compared to ASPB100 and how the pores can easily store the staining agent during application, compromising its removal [38,39].

Borselino et al. [40] produced distinct artificial stones by changing their resin content and also evaluated the influence on the mechanical properties. The authors observed that as the resin content increases, the resistance to staining increases, since the resin has the function of filling all empty spaces on the material’s surface.

## 4. Conclusions

- An innovative artificial stone was developed, using quartz and blast furnace dust waste, demonstrating the efficiency of the incorporation of this waste in the artificial stone’s development. Two binders, pure epoxy (ASPB100) and a mixture of 70 wt% epoxy and 30 wt% cashew nut shell oil (CNLS) (ASPB7030), were used.
- Based on the ternary diagram developed in the experimental numerical modeling grid simplex, the composition that displayed the highest vibrated density, and thus the highest packing, was chosen to produce the stones. These data were authenticated using ANOVA and the Tukey test.
- Both artificial stones presented satisfactory physical indices, with ASPB100 presenting lower porosity and water absorption, making it possible to be applied in humid environments such as kitchens and bathrooms.
- ASPB7030 and ASPB100 displayed 31.0 ± 2.5 MPa and 25.0 ±1.5 MPa mechanical strength in three-point bending, thus classifying them as stones of very high strength (suitable to be applied as coating) and corroborating the porosity results.
- Amsler wear tests indicated that ASPB100 with 0.84 mm ± 0.01 thickness reduction can be applied in floors of high traffic areas, while ASPB7030 with 2.09 mm ± 0.27 thickness reduction can be used for medium traffic ones.
- SEM micrographs proved the physical index and mechanical property results, displaying the optimal load–matrix interaction for both developed stones. ASPB7030 had particles detached from the fractured region, which could be associated with its lower content of epoxy resin. As such, the agglutination of particles in ASPB7030 was not as efficient as in ASPB100.
- The resistance to staining indicated that both stones are not easily stained, except for the red staining agent, which remained in ASPB7030 even after all cleaning steps.
- Both artificial stones produced are an economically viable and sustainable alternatives. ASPB7030 stands out for having two wastes (CNSL and blast furnace dust waste) that do not need any prior treatment, which would mean a cost saving. Both demonstrate excellent physical and mechanical properties for civil construction applications.

## Figures and Tables

**Table 1:** Chemical composition of blast furnace dust waste.

Formula	Concentration (%)
Fe_2_O_3_	74.25
SiO_2_	9.02
CaO	6.27
Al_2_O_3_	2.86
K_2_O	2.52
SO_3_	1.59
Cl	1.41
MgO	0.74
ZnO	0.67
MnO	0.26
TiO_2_	0.21
P_2_O_5_	0.18
SrO	0.03

**Table 2:** Vibrated density of the blast furnace dust quartz sand sample.

Mixture	Coarse (%)	Medium (%)	Fine (%)	Vibrated Density (g/cm^3^)
1	100%	0%	0%	1.53 ± 0.02
2	0%	100%	0%	1.75 ± 0.02
3	0%	0%	100%	0.84 ± 0.02
4	50%	50%	0%	1.88 ± 0.01
5	50%	0%	50%	1.54 ± 0.03
6	0%	50%	50%	1.42 ± 0.01
7	33.33%	33.33%	33.33%	1.72 ± 0.01
8	66.66%	16.66%	16.66%	1.83 ± 0.02
9	16.66%	66.66%	16.66%	1.75 ± 0.01
10	16.66%	16.66%	66.66%	1.34 ± 0.01

**Table 3:** ANOVA test results on the CRD of vibrated density (*p* ≤ 0.05).

FV	GL	SQ	QM	F
Treatment	9	2.5960	0.2884	1185.4018
Waste	20	0.0049	0.0002	
Total	29	2.6009		

Conclusion: If F calculated > F tabulated, there is a statistical difference. F tabulated = 2.39.

**Table 4:** Tukey test for contrasting vibrated density averages (*p* ≤ 0.05).

Treatment	Average	Tukey Test *
4	1.88	A
8	1.83	AB
2	1.75	BC
9	1.75	BC
7	1.72	BC
5	1.54	CD
1	1.53	CD
6	1.42	E
10	1.34	F
3	0.84	G

* Averages followed by the same letter did not differ from each other at the 5% probability level in the Tukey test.

**Table 5:** Compositions of resin and agglomerates used in the development of ASPB7030 and ASPB100.

	ASPB7030
Agglomerates—82%	Resins—18%
67% coarse particles—quartz sand	70%—epoxy
17% medium particles—quartz sand	30% CNSL
16% fine particles—siderurgic waste	
	ASPB100
	100%—epoxy

**Table 6:** Physical properties of the ASPB100 and ASPB7030 artificial stones.

Artificial Stone	Density (g/m^3^)	Water Absorption (%)	Apparent Porosity (%)
ASPB100	2.29 ± 0.05	0.07 ± 0.02	0.14 ± 0.05
ASPB7030	2.21 ± 0.02	0.54 ± 0.20	1.19 ± 0.47

**Table 7:** Thickness reduction in the artificial stones (ASPB100 and ASPB7030) developed after wear tests.

Specimen	Wear Thickness Reduction (mm)	
	500 m	1000 m
ASPB100	0.37 ± 0.01	0.84 ± 0.01
ASPB7030	0.88 ± 0.15	2.09 ± 0.27

## Data Availability

Not applicable.
